# Towards a comprehensive, person-centred assessment of health literacy: translation, cultural adaptation and psychometric test of the Dutch Health Literacy Questionnaire

**DOI:** 10.1186/s12889-020-09963-0

**Published:** 2020-12-02

**Authors:** Jany Rademakers, Geeke Waverijn, Mieke Rijken, Richard Osborne, Monique Heijmans

**Affiliations:** 1 Nivel, Netherlands Institute for Health Services Research, PO Box 1568, 3500 BN Utrecht, The Netherlands; 2 CAPHRI (Care and Public Health Research Institute), Department of Family Medicine, Maastricht University, PO Box 616, 6200 MD Maastricht, The Netherlands; 3 Centre for Global Health and Equity, Faculty of Health, Arts and Design, Swinburne University of Technology, Melbourne, Australia

**Keywords:** Health literacy, Measurement, Health literacy questionnaire, Translation, Psychometrics

## Abstract

**Background:**

Many health literacy instruments focus on reading skills, numeracy and/or information processing aspects only. In the Netherlands, as in other countries, the need for a comprehensive, person-centred measure of health literacy was observed and consequently the decision was made to translate the Health Literacy Questionnaire (HLQ) into Dutch. The HLQ has nine health literacy domains covering people’s experiences and skills. This research sought to translate, culturally adapt and psychometrically test the HLQ.

**Methods:**

The translation and adaptation was done using a systematic approach with forward translation guided by item intents, blind back translation, and a consensus meeting with the developer. The Dutch version of the HLQ was applied in a sample of non-hospitalized, chronically ill patients. Descriptive statistics were generated to describe mean, standard deviation and floor and ceiling effects for all items. A Confirmatory Factor Analysis (CFA) model was fitted to the data. Scores on the nine domains of the HLQ were compared across demographic and illness characteristics as a form of known-groups validity. Psychometric analyses included Cronbach’s alpha, item-rest and item-remainder correlations.

**Results:**

Using CFA, the Dutch HLQ psychometric structure was found to strongly align with the hypothesised (original) nine independent domains of the English version. The nine scales were found to be highly reliable (all scales had alpha between 0.83 and 0.94). Six of the nine HLQ-scales had items that show ceiling-effects. There were no ceiling effects present at the scale level. Scores on the scales of the HLQ differed according to demographic and illness characteristics: people who were older, lower educated and living alone and patients with multiple chronic diseases generally scored lower.

**Conclusions:**

The Dutch version of the HLQ is a robust and reliable instrument that measures nine different domains of health literacy. The questionnaire was tested in a sample of chronically ill patients, and should be further tested in the general population as well as in different disease groups. The HLQ is a major addition to currently available instruments in the Netherlands, since it measures health literacy from a multi-dimensional perspective and builds on patients’ experiences and skills.

## Background

Health literacy is defined by the World Health Organisation as “the cognitive and social skills which determine the motivation and ability of individuals to gain access to, understand and use information in ways which promote and maintain good health.” [1]. Health literacy skills are an important asset for people to find and understand health information, and to take control and responsibility over their health. Furthermore, health literacy is also an important determinant of a person’s ability to recognize health promoting opportunities, access and engage with practitioners and health services and participate in health debates and decision making.

The skills and competencies needed to be a health literate person differ across applications of the concept and these have resulted in a range of definitions [2, 3]. Health literacy began as a notion that concentrated primarily on people’s ability to read and understand health-related information. The concept now includes numerous other factors that relate to what people and communities need to make effective decisions about health for themselves, their families and their communities. Apart from being able to read and to understand information, health literacy is also about the capacity to act [4]. And although health literacy is commonly defined as an individual trait there is also a growing consensus that health literacy does not depend on the skills of individuals alone. Increasingly health literacy is seen as the product of individuals’ capacities and the health literacy–related demands and complexities of the health care system [5, 6]. Governments, health and community services, consumer groups and researchers increasingly recognize their responsibilities to respond appropriately and effectively to the health literacy needs of the consumers they serve and represent and that system changes are needed to align health care demands better with people’s skills and abilities.

Although the conceptualization of health literacy has evolved over time, a great deal of the existing research has been based on instruments that quantify health literacy exclusively or mainly as health-related reading and writing skills (REALM, TOFHLA, Set of Brief Screening Questions), numeracy (Newest Vital Sign) and comprehension of medical concepts (SAHL) [7–11]. A comprehensive conceptual framework is lacking in these measures of health literacy. The HLS-EU questionnaire, developed by Sørensen et al., was based on a theoretical model, but still focussed predominantly on cognitive skills and information processing [3].

A well-structured conceptual model is important when developing and testing a measurement tool. Such conceptual models can be generated through qualitative methods and mixed methods processes such as concept mapping and they can help to ensure that the content of an instrument is a comprehensive representation of a person’s lived experience in a particular setting [12]. With respect to health literacy instruments, the concept has been evolving, they have been used for a range of purposes in a wide range of settings. The use of a strong conceptual model can assist researchers to determine if a candidate tool contains all relevant elements (i.e., construct representation) or whether elements are missing (construct under representation) in the measures [12, 13]. In addition, most of these instruments have been developed by researchers or health care professionals with minimal input of patients or citizens. The inclusion of patients and citizens in the instrument development process, however, is a crucial factor to ensure content validity [14]. Osborne et al. concluded that the aforementioned “measures of health literacy … fail to capture the full breadth of ideas embodied in definitions of health literacy and they have also been shown to have substantive psychometric weaknesses” [15–17]. For these reasons, research was undertaken to develop a new measurement instrument, the Health literacy Questionnaire (HLQ) [15]. Health literacy was broadly defined as a person’s ability to understand, access and use health information and health services. The HLQ was developed from patient’s and professional’s perspectives using concept mapping. The seeding question in the concept mapping was “What does a person need to be able to understand, get and use the health information and services they need?”. Concept mapping includes qualitative (e.g., group consensus) and quantitative components (multidimensional scaling and cluster analysis) to assist in the generation of a conceptual framework grounded in participants’ daily experiences. This process derived a conceptal model with nine separtate constructs. The constructs cover aspects of information management, self-reported personal skills and perceived social support. The skills vary from the ability to understand and critically appraise health information, to being able to actively engage with healthcare providers and navigate the healthcare system. In summary, the HLQ was developed using a validity-driven approach including in-depth grounded consultations, cognitive interviews to generate a conceptual model for testing using psychometric analyses [18]. The HLQ has been found to be highly relevant to a wide range of stakeholders and has strong and reproducible psychometric properties [15].

The relevance of a multi-dimensional health literacy measurement instrument as the HLQ to health care and public health is that it generates a broader, in-depth profile of the health literacy level of individuals and populations. In this way, the HLQ may capture both strengths and weaknesses, which are pertinent to clinicians, public health practitioners and health planners. For example, an individual or community may have very poor health-related reading or numeracy ability (and thus be classified as having low health litery on this single dimension) yet they have had strong social support for their health issues and excellent ability to communicate with their doctors, who are responsive to their needs. The use of a multi-dimensional measure of health literacy permits more nuanced and real-world interpretation of the health literacy situation of individuals and communites. Furthermore, the profiles of strengths and weaknesses can be used as specific input for tailored care and interventions. For example, individuals and populations who score relatively low on the constructs ‘understanding and appraising health information’ but relatively high on ‘social support for their health’ may benefit from interventions based on provision of supporting people to interpret information through the help of a relative or friend. Conversley, a different health literacy intervention strategy would need to be devised in the target individuals did not have access to friends or family to support them.

The HLQ is now one of the mostly widely used health literacy tools in public health and health services research and has been translated into over 30 languages including Danish, Slovakian, German, Norwegian and French [19–23]. In the Netherlands, a comprehensive, person-centered measure of health literacy was lacking, consequently we decided to translate the HLQ into Dutch. Since the HLQ is used around the world, a Dutch version would also be useful for international comparative research. In this article we describe the translation, cultural adaptation and psychometric testing of the HLQ.

## Methods

### Translation

For the translation and cultural adaptation of the HLQ, a systematic approach was followed [24]. This method -that follows WHO guidelines- includes the following steps: (1) forward translation (guided by detailed descriptions of the intent of each item), (2) expert panel meeting, (3) backward translation, (4) pre-testing/cognitive interviewing and (5) consensus on the final version.

(1) Forward translation: Two independent Dutch translators performed the forward translation. The expert panel included one of the translators, three Dutch researchers (JR, MR, MH), two lay persons and one of the developers (RO).

(2) Expert panel meeting: The expert panel discussed discrepancies between the two translations. After the expert panel meeting, a translation was agreed upon.

(3) Backward translation: The instrument was then translated back into English by a third, independent translator. There were only a few minor textual discrepancies between the back translation and the original instrument, which were corrected.

(4) Pre-testing/cognitive interviewing: Pre-testing was done through 15 individual cognitive interviews in order to check whether the intent of the translated items were equivalent to the original. In this way, we determined whether the translated questionnaire was culturally appropriate in the Dutch context. During the cognitive interviews participants were asked reflective questions to gain insights into their reasoning and decision-making process when answering the questions. Respondents for example were asked ‘What were you thinking about when you were answering that question?’ This process elicited the cognitive process behind the answers. After completion, the researcher undertook further specific probing related to items that respondents may have had problems understanding or answering. Probing questions were ‘Why did you find this question difficult to answer?’ and ‘What do you think the researchers want to know here?’ In this way, we gained insight into the readability and clarity of the items and we assessed the meaning and interpretation of the individual questions, to establish if there were any problems with respect to the cultural congruency of the items. Such validity issues can arise when a). the questionnaire is translated from English to Dutch and some words might have a different meaning or connotation than intended in the original instrument and b). the HLQ is developed in another country (Australia) and aspects of the health care system might differ from the situation in the Netherlands.

No problems regarding the translation and/or cultural sensitivity arose for any items during the cognitive interviews. Only a few minor alterations in wording were made on the basis of the interviews.

(5) Consensus on the final version: after this procedure, the research team approved of the final version that was to be tested. A version of the Dutch translation of the HLQ is available upon request from HLQ-info@swin.edu.au, as is the original instrument.

### Sample

We sought to determine if the HLQ was a suitable health literacy research tool in one of our main research areas (patients with a chronic illness). The HLQ was therefore sent to participants of the ‘National Panel of people with Chronic illness or Disability’ (NPCD). The NPCD is a national prospective panel survey that focuses on the consequences of chronic illness and disability from the patient’s perspective. It consists of > 4000 people with a chronic illness or physical disability. Panel members fill in questionnaires at home twice a year. On the NPCD website, a full description of the panel and the selection procedures is provided [25]. In summary, NPCD panel members with a chronic illness:

- are recruited from random samples of Dutch general practices;

- have a diagnosis of a somatic chronic disease by a medical practitioner and know their diagnosis;

- are not terminally ill (life expectancy > 6 months);

- are 15 years and older, with a sufficient mastery of the Dutch language;

- are not institutionalized and are mentally capable of participating in a survey study.

In addition, panel members with a physical disability are recruited through national population surveys conducted by the Central Statistics Office in the Netherlands (CBS).

For the purpose of this study, we selected panel members who had been diagnosed with a chronic disease. The questionnaire was sent to a sample of to 2375 panel members, of which 1993 (84%) returned the questionnaire. Non-response analysis revealed that respondents were older than non-responders (mean age 63 versus 58 years) and responders more often had comorbidity than non-responders (53% versus 45% respectively). There were no differences between responders and non-responders in sex and educational level.

### Measures

#### Health literacy

The HLQ measures nine domains of health literacy (44 questions) [15]. The domains are: (1) feeling understood and supported by healthcare providers (4 items); (2) having sufficient information to manage my health (4 items); (3) actively managing my health (5 items); (4) social support for health (5 items); (5) critical appraisal of health information (5 items); (6) ability to actively engage with healthcare providers (5 items); (7) navigating the healthcare system (6 items); (8) ability to find good health information (5 items); and (9) understand health information well enough to know what to do (5 items). For the list of questions of the HLQ see Osborne et al. [15]. Approximately half (23) of the 44 questions (scales 1–5) have four-point Likert response categories: ‘Strongly disagree’, ‘Disagree’, ‘Agree’, ‘Strongly agree’. The remaining 21 questions (scales 6–9) have a five-point Likert response categories: ‘Cannot do’, ‘Very difficult’, ‘Quite difficult’, ‘Quite easy’, ‘Very easy’.

#### Demographic and illness characteristics

In the analyses, we included a number of demographic and illness characteristics, namely: sex; age; educational level, coded as either low (no education to lowest high school degree), intermediate (vocational training to high school degrees), high (university of applied sciences degree and university degree); living situation (living alone or living together with a partner and/or children); the first diagnosed chronic disease or ‘index disease’ (including cardiovascular diseases, cancer, respiratory diseases, diabetes, musculoskeletal diseases, neurological diseases, digestive diseases and a category ‘other’) as reported by the general practitioner; number of chronic diseases (ranging from 1 to ‘3 or more’) and illness duration (in years since diagnosis).

### Statistical analysis

To assist with comparing the results of our study with the original development study [15] and four other translation and validation studies [19–23] we used similar statistical protocols. The main research questions that guided the analyses were: (1) does the original hypothesised model of the HLQ with nine subscales also exist in the Dutch data?, (2) are the individual items robust?, and 3) are the subscales internally consistent? Descriptive statistics were generated to describe mean, standard deviation and floor and ceiling effects for all items. Floor and ceiling effects were considered to be present if > 15% of respondents scored the worst or the best possible score [26]. Internal consistency for the nine scales was evaluated using Cronbach’s alpha, item-total correlations and item-remainder correlations based on a polychoric correlation matrix, appropriate for the analysis of ordinal data [27]. We conducted Confirmatory Factor Analysis (CFA) with LISREL 8.70 (Scientific Software International, Lincolnwood, IL) to evaluate the extent to which the items loaded on the hypothesised scales and the degree to which the scales were correlated. A nine-factor CFA-model (with no factor cross-loadings and no correlated item residuals) was fitted to the data, using the diagonally weighted least squares (DWLS) approach available in LISREL. The DWLS method is recommended for ordinal data [28]. The DWLS is a robust weighted least squares method and is based on the polychoric correlation matrix of the variables included in the analysis. We examined the model fit with the Non-Normed Fit Index (NNFI), the comparative fit index (CFI), the standardize root mean square residual (SRMR), and the root mean square measure of approximation (RMSEA). NNFI and CFI values ≥0.95, SRMR ≤0.08 and RMSEA values ≤0.06 were considered indicative of good model fit [29]. We used a cutoff-value of 0.4 to define acceptable factor loadings [30].

Scores on the nine scales of the HLQ were described by demographic and illness characteristics. A scale score was regarded as missing if more than 50% of the items in a scale were missing. To assess whether the HLQ scale scores discriminate between subgroups of people with chronic disease, we tested for differences between sex, age and educational level living situation, type of disease, number of chronic conditions using t-tests and ANOVA’s. For the relationship between illness duration and HLQ scale scores we looked at bivariate correlations.

## Results

Participant characteristics are shown in Table 1. Approximately half of the people in our sample were female with a mean age of 63 years. Almost one third of the respondents (32%) had low education and three quarters lived in a household with a partner and/or children (76%). Half of the respondents (53%) had more than one medically diagnosed chronic disease and the mean illness duration was 13 years.

**Table 1:** Descriptive statistics of sample of people with chronic illness (*n* = 1993)

		**Percent**
Sex	Male	47.1
	Female	52.9
		**Mean (sd)**
Age in years		63.2 (13.8)
		**Percent**
Age in years	15 t/m 64	46.9
	65 t/m 74	33.1
	75 and older	20.0
Education	Low	31.8
	Intermediate	43.3
	High	25.0
Living situation	Living alone	24.5
	Living together	75.5
First diagnosed chronic disease	Cardiovascular disease	14.2
	Respiratory disease	33.9
	Musculoskeletal disease	9.4
	Cancer	13.5
	Diabetes	10.0
	Neurological disease	4.5
	Digestive disease	3.7
	Unspecified other disease	10.8
Presence and number of chronic illnesses	1	46.8
	2	30.7
	3 or more	22.5
		**Mean (sd)**
Illness duration in years		12.8 (9.9)

### Structural validity

Confirmatory Factor Analysis showed good fit indices for a nine-factor model of the HLQ-scales. CFI (0.990), NNFI (0.989), RMSEA (0.0537) and SRMR (0.068) were all within the pre-specified cut-off criteria. Standardized factor loadings ranged from 0.48 to 0.98 (Table 2). Almost all items had satisfactory factor loadings. Only five items had factor loadings lower than 0.70. The item with the lowest standardized factor loading was ‘I spend quite a lot of time actively managing my health’ on scale 4 ‘Actively managing my health’ (0.48). Six items had a reliability (defined as the variation explained by their corresponding factor) lower than 0.50, including the item mentioned above (Table 2).

**Table 2:** Factor loadings of the nine-factor confirmatory factor model (*n* = 1993)

	Factor loading	Error variance^a^	T-statistic^b^	R^**2**^
**1. Feeling understood and supported by healthcare providers**				
I have at least one healthcare provider who …	0.72	0.49	26.22	0.52
I have at least one healthcare provider I can …	0.81	0.34	28.99	0.66
I have the healthcare providers I need …	0.98	0.05	51.11	0.95
I can rely on at least one …	0.88	0.23	43.49	0.77
**2. Having sufficient information to manage my health**				
I feel I have good information about health …	0.80	0.36	29.89	0.64
I have enough information to help me deal …	0.82	0.33	33.19	0.67
I am sure I have all the information I …	0.80	0.37	31.06	0.63
I have all the information I need to …	0.86	0.26	40.61	0.74
**3. Actively managing my health**				
I spend quite a lot of time actively managing …	0.48	0.77	14.36	0.23
I make plans for what I need to do to be …	0.73	0.46	24.67	0.54
Despite other things in my life, I make time …	0.77	0.41	25.27	0.59
I set my own goals about health and fitness …	0.93	0.13	31.63	0.87
There are things that I do regularly …	0.82	0.33	31.85	0.67
**4. Social support for health**				
I can get access to several people who …	0.87	0.24	37.62	0.76
When I feel ill, the people around me really …	0.70	0.51	25.58	0.49
If I need help, I have plenty of people I..	0.89	0.21	45.56	0.79
I have at least one person … .	0.63	0.61	19.21	0.39
I have strong support from …	0.73	0.47	28.86	0.53
**5. Appraisal of health information**				
I compare health information from different …	0.66	0.57	21.01	0.44
When I see new information about health, I …	0.51	0.74	14.81	0.26
I always compare health information from …	0.66	0.57	21.90	0.43
I know how to find out if the health …	0.98	0.03	40.41	0.97
I ask healthcare providers about the quality …	0.68	0.54	20.33	0.46
**6. Ability to actively engage with healthcare providers**				
Make sure that healthcare providers understand..	0.85	0.28	56.08	0.72
Feel able to discuss your health concerns with a..	0.85	0.27	72.35	0.73
Have good discussions about your health …	0.85	0.29	51.85	0.71
Discuss things with healthcare providers …	0.89	0.21	85.95	0.79
Ask healthcare providers to get …	0.92	0.16	87.85	0.84
**7.Navigating the healthcare system**				
Find the right healthcare...	0.83	0.31	58.45	0.69
Get to see the healthcare provider you need to …	0.81	0.35	48.22	0.65
Decide which health care provider you need …	0.86	0.26	66.01	0.74
Make sure you find the right place to get …	0.87	0.25	59.68	0.75
Find out what healthcare services you are …	0.76	0.43	43.57	0.57
Work out what is the best care for you …	0.86	0.26	65.67	0.74
**8. Ability to find good health information**				
Find information about health problems …	0.85	0.27	60.87	0.73
Find health information from several …	0.86	0.26	72.94	0.74
Get information about health so you are …	0.90	0.19	69.23	0.81
Get health information in words you …	0.86	0.27	66.92	0.73
Get health information by yourself …	0.88	0.23	70.12	0.77
**9. Understanding health information well enough to know what to do**				
Confidently fill medical forms in the correct …	0.85	0.28	46.28	0.72
Accurately follow the instructions from …	0.83	0.31	46.71	0.69
Read and understand written health …	0.81	0.34	52.60	0.66
Read and understand all the information on …	0.78	0.39	47.34	0.61
Understand what healthcare providers are …	0.91	0.18	73.38	0.82

^a^Proportion of variance in the measure not attributable to the latent factor

^b^The ratio of each parameter estimate and its standard error is distributed as a t-statistic. All t-statistics are significant at *P* < 0.001

Correlation between factors showed a clear discrimination between the disagree/agree scales (the range of inter-factor correlations is 0.44 to 0.71) (Table 3). Clear discrimination was less apparent for the scales options with the cannot do/very easy response: the range of inter-factor correlations was 0.89 to 0.96.

**Table 3:** Correlation between latent variables (*n* = 1.993)

	1.	2.	3.	4.	5.	6.	7.	8.	9.
1.Feeling understood and supported by health care providers	1								
2.Having sufficient information to manage my health	0.69	1							
3.Actively managing my health	0.45	0.59	1						
4.Social support for health	0.64	0.68	0.44	1					
5.Appraisal of health information	0.49	0.65	0.71	0.47	1				
6.Ability to actively engage with healthcare providers	0.62	0.70	0.32	0.57	0.47	1			
7.Navigating the health care system	0.54	0.73	0.33	0.55	0.49	0.96	1		
8.Ability to find good health information	0.44	0.73	0.35	0.50	0.57	0.89	0.96	1	
9.Understanding health information well enough to know what to do	0.43	0.62	0.32	0.43	0.44	0.89	0.92	0.93	1

### Item analysis and reliability

Table 4 shows the means and standard deviations for items, the presence of floor and ceiling effects, item correlations and alpha’s for the scales after item removal. Six of the nine HLQ-scales had items that show ceiling-effects. Ceiling effects were mainly present in the scales with the difficulty response options (scales 6 to 9). There were no ceiling effects present at the scale level (data not shown).

**Table 4:** Descriptive statistics of items and internal consistency of scales (*n* = 1993)

**1. Feeling understood and supported by healthcare providers – Alpha = 0.91 (*N*** **= 1895)**					
	Mean (sd)	Floor/ceiling effects	Item-total correlation	Item-remainder correlation	Alpha if item is deleted
I have at least one healthcare provider who …	3.0	Ceiling	0.88	0.77	0.89
I have at least one healthcare provider I can …	3.0	No	0.90	0.82	0.87
I have the healthcare providers I need …	2.9	No	0.85	0.73	0.90
I can rely on at least one …	3.0	No	0.92	0.85	0.86
**2. Having sufficient information to manage my health – Alpha = 0.89 (*N*** **= 1895)**					
I feel I have good information about health …	3.0	No	0.82	0.67	0.89
I have enough information to help me deal …	3.0	No	0.88	0.79	0.85
I am sure I have all the information I …	2.8	No	0.89	0.79	0.85
I have all the information I need to …	2.9	No	0.89	0.80	0.85
**3. Actively managing my health – Alpha = 0.86 (*N*** **= 1897)**					
I spend quite a lot of time actively managing …	2.6	No	0.72	0.56	0.86
I make plans for what I need to do to be …	2.9	No	0.83	0.72	0.82
Despite other things in my life, I make time …	2.8	No	0.84	0.74	0.81
I set my own goals about health and fitness …	3.0	No	0.75	0.60	0.85
There are things that I do regularly …	2.9	No	0.86	0.77	0.81
**4. Social support for health – Alpha = 0.86 (*N*** **= 1913)**					
I can get access to several people who …	2.9	No	0.84	0.74	0.82
When I feel ill, the people around me really …	2.7	No	0.75	0.60	0.86
If I need help, I have plenty of people I..	2.9	No	0.87	0.78	0.81
I have at least one person … .	3.1	Ceiling	0.72	0.56	0.87
I have strong support from …	2.9	Ceiling	0.85	0.76	0.82
**5. Appraisal of health information– Alpha = 0.83 (*N*** **= 1898)**					
I compare health information from different …	2.7	No	0.80	0.67	0.78
When I see new information about health, I …	2.6	No	0.79	0.65	0.79
I always compare health information from …	2.6	No	0.86	0.77	0.75
I know how to find out if the health …	2.8	No	0.69	0.50	0.83
I ask healthcare providers about the quality …	2.5	No	0.72	0.55	0.82
**6. Ability to actively engage with healthcare providers– Alpha = 0.94 (*N*** **= 1882)**					
Make sure that healthcare providers understand..	3.9	No	0.87	0.79	0.93
Feel able to discuss your health concerns...	4.0	Ceiling	0.91	0.85	0.92
Have good discussions about your health …	4.0	Ceiling	0.88	0.82	0.92
Discuss things with healthcare providers …	4.0	Ceiling	0.89	0.83	0.92
Ask healthcare providers to get …	4.0	Ceiling	0.90	0.85	0.92
**7. Navigating the healthcare system– Alpha = 0.93 (*N*** **= 1875)**					
Find the right healthcare...	3.9	Ceiling	0.85	0.78	0.92
Get to see the healthcare provider you need to..	4.0	Ceiling	0.83	0.75	0.92
Decide which health care provider you need …	3.9	Ceiling	0.89	0.83	0.91
Make sure you find the right place to get …	4.0	Ceiling	0.89	0.83	0.91
Find out what healthcare services you are …	3.6	No	0.82	0.74	0.92
Work out what is the best care for you …	3.8	No	0.87	0.80	0.91
**8. Ability to find good health information– Alpha = 0.93 (*N*** **= 1881)**					
Find information about health problems …	4.0	Ceiling	0.88	0.82	0.92
Find health information from several …	3.9	Ceiling	0.91	0.86	0.91
Get information about health so you are …	3.9	No	0.89	0.82	0.92
Get health information in words you …	4.1	Ceiling	0.84	0.75	0.93
Get health information by yourself …	3.9	Ceiling	0.90	0.85	0.91
**9. Understanding health information well enough to know what to do– Alpha = 0.91 (*N*** **= 1884)**					
Confidently fill medical forms in the correct …	4.0	Ceiling	0.86	0.78	0.88
Accurately follow the instructions from …	4.0	Ceiling	0.78	0.66	0.91
Read and understand written health …	4.0	Ceiling	0.87	0.79	0.88
Read and understand all the information on …	4.0	Ceiling	0.85	0.77	0.89
Understand what healthcare providers are …	4.1	Ceiling	0.90	0.84	0.87

Cronbach’s alpha indicated good to excellent internal consistency for all of the nine scales (the lowest alpha was 0.83 and the highest 0.94). Deletion of an item would not lead to useful improvements in internal consistency in any scale.

### Relationship with demographic and illness characteristics

Scale scores differed according to demographic characteristics (Table 5). Differences with regard to sex were only found on two scales; women scored higher on ‘4. Actively managing my health’ and ‘9. Understanding health information well enough to know what to do’. People who were older and who had low education reported lower scores on the majority of HLQ scales than people who were younger and with higher educational attainment. People who lived alone scored somewhat lower on all scales of the HLQ than people who live with a partner and/or children, with exception of the scales ‘1. Feeling understood and supported by health care providers’, ‘2. Having sufficient information to manage my health’ and ‘3. Actively managing my health.

**Table 5:** Scores on scales of the HLQ by demographic characteristics

	Sex (***n*** = min. 1881)		Age (***n*** = min. 1881)			Educational (***n*** = min. 1686)			Living Situation (***n*** = min 1700)	
	Men (ref)	Women	15 t/m 64 (ref)	65 t/m 74	75 +	Low (ref)	Middle	High	Alone (ref)	Together
1.Feeling understood and supported by health care providers	3.0	3.0	3.0	3.0	3.0	2.9	3.0	3.0	3.0	3.0
2.Having sufficient information to manage my health	2.9	2.9	2.9	2.9	2.9	2.9	2.9**	3.9**	2.9	2.9
3.Actively managing my health	2.8	2.9**	2.9	2.8	2.8**	2.8	2.9	2.9	2.8	2.9
4.Social support for health	2.9	2.9	2.9	2.9	2.9	2.9	2.9	2.9	2.8	2.9***
5.Appraisal of health information	2.6	2.7	2.7	2.6	2.6**	2.6	2.6	2.7***	2.6	2.7*
6.Ability to actively engage with healthcare providers	4.0	4.0	4.0	3.9**	3.9***	3.8	4.0***	4.1***	3.9	4.0***
7.Navigating the health care system	3.9	3.9	4.0	3.9**	3.7***	3.7	3.9***	4.0***	3.8	3.9***
8.Ability to find good health information	3.9	3.9	4.1	3.9***	3.7***	3.7	4.0***	4.1***	3.8	3.9***
9.Understanding health information well enough to know what to do	4.0	4.1**	4.1	4.0***	3.9***	3.8	4.1***	4.3***	4.0	4.1**

***P < 0.001, **P < 0.01, *p < 0.05

There were also some differences in scores on the HLQ with regard to illness characteristics. People with multiple chronic diseases scored lower on a number of scales (Table 6). On scales ‘7. Navigating the health care system’, ‘9. Understanding health information well enough to know what to do’ and ‘8. Ability to find good health information’, people with three or more chronic diseases scored significantly lower than people with one chronic disease. People with two chronic diseases scored lower than people with one chronic disease on the scales ‘9. Understanding health information well enough to know what to do’ and ‘8. Ability to find good health information’.

**Table 6:** Scores on scales of the HLQ by number of diseases

	Number of chronic conditions (***n*** = min. 1709)		
	1(ref)	2	3 or more
1.Feeling understood and supported by health care providers	2.97	2.94	2.99
2.Having sufficient information to manage my health	2.93	2.91	2.90
3.Actively managing my health	2.86	2.84	2.85
4.Social support for health	2.92	2.91	2.88
5.Appraisal of health information	2.65	2.64	2.60
6.Ability to actively engage with healthcare providers	4.0	3.94	3.92
7.Navigating the health care system	3.91	3.85	3.81**
8.Ability to find good health information	3.95	3.86**	3.80***
9.Understanding health information well enough to know what to do	4.09	4,01**	3.96***

***P < 0.001, **P < 0.01, *p < 0.05

There were also some minor differences with respect to index disease (data not shown) although these differences did not follow a consistent pattern across diseases. In general patients with diabetes had a somewhat higher score then patients with other chronic diseases. Based on bivariate correlations, illness duration was unrelated to the level of health literacy skills.

## Discussion

In this article we have described the translation, cultural adaptation and psychometric testing of the Dutch version of the HLQ. The translation and adaptation was done using a systematic approach that follows WHO guidelines and was provided by the developers of the original instrument. The same procedure was used for other translations of the HLQ [19–23].

No problems regarding the translation and/or cultural sensitivity arose during cognitive interviews. The Dutch version of the HLQ was tested in a sample of non-hospitalized patients with at least one chronic illness and found to be a robust instrument. The Dutch HLQ has a strong psychometric structure as demonstrated by confirmatory factor analyses which indicated good model fit. The psychometric analyses confirmed that the Dutch version of the HLQ is consistent with the original hypothesised nine distinct domains of health literacy. The internal consistency of all scales of the Dutch HLQ was good to excellent. Some of the scales have high correlations. As already suggested by Osborne et al. [15], higher order factors may be present. Another possible explanation is that some scales are causal of others. Six of the nine HLQ-scales had items that show ceiling-effects. This may either imply that these items are not difficult enough and therefore they do not discriminate in this population, or that the population under study overall had high competency levels regarding these aspects or behaviours. No ceiling effects were present at the scale level.

There were differences in the scores on the HLQ according to age, educational level and living situation, especially in the scales that measure people’s self-perceived skills (scales 6–9). Older patients score lower compared to younger patients, people with a low education level score lower compared to people with intermediate or higher education levels and people that were living alone scored lower on certain skills than people living with a partner or children.

Our results are in line with the psychometric outcomes found in Denmark [19] and Germany [21]. These researchers also found evidence for a nine factor structure and good to excellent internal consistency of the separate scales. Like in our study both studies reported some high correlations between some of the factors.

Given the results of the psychometric tests, the Dutch version of the HLQ can be considered a good replication of the original English questionnaire. It measures health literacy from a multi-dimensional perspective and captures a wide range of patients experiences and skills and would reveal individuals and groups health literacy strengths and weaknesses. It therefore adds to the currently available instruments in the Netherlands, which primarily focus on reading skills, numeracy and/or information processing aspects only.

The sociodemographic differences in the scores of the respondents are in line with recent insights about the distribution of health literacy in the Dutch general population, based on measurement with the HLS-EU 16-item questionnaire [31]. However, in that same study men had significantly lower health literacy scores compared to women [31]. In the current HLQ study, differences between men and women were marginal. Only on two scales was a significant difference in the favor of women ‘3. Actively managing my health’ and ‘9. Understanding health information well enough to know what to do’. The reason for this might be that the men in our sample are volunteers in a panel study which focuses on health, healthcare, wellbeing and participation of people with a chronic illness or disability and therefore more interested in these topics compared to men in the general population. Furthermore, having a chronic disease is known to increase people’s interest in and knowledge of health and healthcare, in particular around their own illness. Both these explanations (being member of a panel and having one or more chronic diseases) may thus lead to a relatively high level of health literacy skills in the population under study which may account for the ceiling effects in some of the items as well. In another HLQ study involving several groups of health care users, modest or no floor and ceiling effects were found [32]. We therefore consider further investigation into the difficulty level and discriminatory power of the specific items of the Dutch translation of the HLQ important. The results of this study should be compared with further validity testing studies in the general population as well as in different disease groups in the Netherlands. Other analyses (e.g. on measurement invariance, difficulty level and discrimination power of the items) could be used to further validate the HLQ in the Netherlands.

An important strength of the HLQ is that it measures a range of different domains of health literacy. On the basis of these respective components it is possible to construct health literacy profiles of individuals and populations, both in healthcare as well as in public health settings. Knowing the health literacy strengths and weaknesses of their patients or target group allows healthcare providers, organisations and (local) governments to optimize their response strategies. By providing more appropriate and tailored care and support, equity in health outcomes and access will likely be improved. Currently, in different countries, the HLQ is the basis for such targeted efforts in the form of WHO National Health Literacy Demonstration Projects [33]. A disadvantage of the full HLQ is that it is a relatively long questionnaire (44 items). This takes time and effort of participants, and especially for people with low literacy skills, self-administration may be be too demanding. For this group, face-to-face or telephone interviews are an alternative which was a procedure used in the Danish validity testing study [19]. Furthermore, the questionnaire might often be too long to be fully integrated in a standard survey. Since the questionnaire is divided in nine domains, researchers may choose scales as an indicator of specific health literacy skills. Since each of the scales clearly focus on a specific subset of skills, researchers may select the most appropriate scales given their specific research question. This is an advantage over shorter, generic health literacy instruments that attempt to measure the complex concept of health literacy as a singular construct.

### Strengths and limitations

A strength of this study is that it builds upon a well-established line of research. The conceptual model of the HLQ and the original questionnaire are developed on the basis of in-depth grounded consultations, cognitive interviews and extensive psychometric analyses. The protocol that guided the translation and cultural adaptation process, including the item intents, expert meeting and cognitive interviews, helped to ensure that the items in the Dutch version captured the same meaning and difficulty level compared to the original questionnaire.

A limitation of the study is that our sample was drawn from an already existing panel, and that people were thus able to fill out the questionnaire themselves. This may have led to less variation in and possibly higher scores compared with the general population, since all respondents were literate. However, almost one out of three respondents (only) had a low education level. Nevertheless, further testing of the questionnaire in groups who are generally not included in survey studies (such people who have literacy problems or who do not speak the Dutch language fluently) might lead to different conclusions. Also, other modes of administration should be researched, such as face-to-face interviews.

## Conclusions

The Dutch version of the HLQ is a robust and reliable instrument that measures nine different domains of health literacy. The questionnaire was tested in a sample of chronically ill patients, and should be further tested in the general population as well as in different disease groups. The HLQ is a major asset to the set of currently available instruments in the Netherlands, since it measures health literacy from a multi-dimensional perspective and explored patients’ experiences and skills.

## Data Availability

The datasets used and analyzed during the current study are available from the corresponding author on reasonable request.

## Abbreviations

HLQ: Health Literacy Questionnaire
CFA: Confirmatory Factor Analysis
REALM: Rapid Estimate of Adult Literacy in Medicine
TOFHLA: Test of Functional Health Literacy in Adults
NPCD: National Panel of people with Chronic illness or Disability
DWLS: Diagonally weighted least squares
NNFI: Non-Normed Fit Index
CFI: Comparative fit index
SRMR: Standardize root mean square residual
RMSEA: Root mean square error of approximation
SD: Standard deviation
HLS-EU: Health literacy survey Europe
